# Integrating augmented reality into health media campaigns: toward a more behavioral impact economic evaluation

**DOI:** 10.3389/fpubh.2025.1682659

**Published:** 2025-11-03

**Authors:** Mohammed Habes, Amirah ALZahrani, Suhib Y. Bdoor, Amal Hassan Alhazmi, Areej Abdulllah Alfawaz, Razaz Waheeb Attar

**Affiliations:** ^1^Faculty of Mass Communication, Radio & TV Department, Yarmouk University, Irbid, Jordan; ^2^Department of Curricula and Instruction, College of Education, University of Bisha, Bisha, Saudi Arabia; ^3^Department of Management, College of Business Administration, Princess Nourah Bint Abdulrahman University, Riyadh, Saudi Arabia

**Keywords:** augmented reality, health communication, cost-effectiveness analysis, behavioral intention, cross-national comparison, immersive media

## Abstract

This study investigates the efficacy and economic efficiency of augmented-reality (AR)–enhanced health-media campaigns in urban settings of Jordan, the Saudi Arabia. Employing a quasi-experimental, comparative design, 600 adults aged 18–45 were randomly assigned to either an AR intervention—featuring interactive 3D simulations of smoking risks and vaccination mechanisms—or a conventional video/text campaign. Pre- and post-intervention surveys measured cognitive/emotional engagement, behavioral intention, and self-reported health actions, while detailed cost logs enabled incremental cost-effectiveness ratio (ICER) analyses. Results demonstrated that AR immersion significantly elevated presence [*F*(1,594) = 152.07, *p <* 0.001] and time-on-task [*F*(1,594) = 210.33, *p <* 0.001], which in turn produced larger and more durable increases in intention (*η*^2^ = 0.14 for Arm × Time interaction) and actual behavior change [smoking reduction *t*(598) = 20.84, *p <* 0.001; vaccination uptake *χ*^2^(1) = 32.56, *p <* 0.001]. Economic evaluation revealed that AR campaigns achieved lower ICERs (USD 29.50 per unit behavior change) compared to conventional media, with sensitivity analyses confirming robustness. Multi-group moderation analyses confirmed stronger path coefficients and greater cost-efficiency in the Saudi Arabia sample, underscoring the moderating role of technological readiness and cultural factors. These findings affirm AR’s promise as a cost-effective modality for immersive health promotion.

## 1 Introduction

Never before in history did digital innovation and population outreach related to public health ever converge in the way it has done in the 21st century and this has seen a paradigm shift in how populations read, interpret and react to important health information (1). In the context of this change, augmented reality (AR) has been discovered as an effective mechanism to engage learners in learning complicated biomedical ideas in the form of immersive and richly contextualized visualizations to provide a better learning experience and development of understanding. Notwithstanding its potential, the uptake of AR in the mass-communication health campaign is in its infancy period, especially in the Middle East and North Africa (MENA) region, where economic and cultural factors may moderate the adoption, as well the efficacy, of AR. The health-media communication landscape has been totally transformed by high rates of digitalization, which is emphasizing the transformation of one-way and static, passive information dissemination to a more dynamic and user focused interaction. The traditional broadcast and print channels, though they are fundamental have the drawback because they cannot be personalized and cannot facilitate real-time feedback and, therefore, the effects they have on rigid health habits are limited. On the contrary, AR extends the same process by introducing virtual principles to the real-life spaces of users to provide the so-called presence, an immersive performance that is cognitive and emotional engagement superior to that of two-dimensional display sources (2). The efficacy of AR in medical training, surgical assistance, and patient education is proven by experimental works: Barsom et al. (3) demonstrated that including AR simulation in the learning process led to a better improvement in the learning progress, and that Moro et al. (4) revealed a higher retention of anatomy when using AR overlays. Overall, these findings lead to the suggestion that AR may replace the conventional pedagogies and be used as a groundbreaking tool in health awareness programs (4–6). Although success of AR has been reported in controlled educational and professional clinical settings, it does not extend well into mass communication pointed health campaign. Traditional campaigns which anchor mostly on posters, videos, and didactic communications often fail to create lasting change in behavior, especially in certain groups of people who may have a die-hard loyalty or lack interest. Indeed, studies highlight a persistent “intention-behavior gap,” wherein favorable attitudes toward health recommendations fail to manifest as concrete actions (7). Moreover, while AR affords immersive engagement, scant research has systematically evaluated its cost-effectiveness relative to standard media formats, especially within MENA countries characterized by variable technology infrastructure and diverse cultural attitudes toward digital interventions. Consequently, there exists a critical lacuna in evidence regarding whether the incremental investments in AR development and distribution yield proportional gains in health outcomes and economic efficiency (8, 9). This is what this research has proceeded to intense search of comparative behavioral and economical impact of AR-enriched and conventional media campaign within Jordan and Saudi Arabia, attempting to bridge the gaps between presence theoretical process and desired behavioral signal and actual cost effectiveness indicators (2, 7). To ground the intervention in a robust theoretical framework, this study integrates three complementary perspectives. First, the Theory of Planned Behavior (7) provides a foundation for explaining how attitudes, subjective norms, and perceived behavioral control jointly shape behavioral intentions, which then predict health actions across diverse contexts. Second, the concept of presence in immersive environments (2) underscores how augmented reality (AR) heightens the sense of “being there,” thereby strengthening cognitive elaboration and emotional resonance. Immersion is demonstrated to support the retention and acquisition of skills, as evidenced by the empirical studies of medical training in Europe (3) and anatomy training in Australia (4), which are the same objectives as those of public-health campaigns. Lastly, behavioral economics and nudge theory can help us understand that making small adjustments to the decision environment can influence health decisions without forcing them (10). Plausible examples include their application in Sweden in enhancing the use of vaccination (11) and the United States in reducing smoking (12). Combined, these frameworks explain the processes by which AR will be supposed to increase engagement, intention, and eventually encourage healthier behaviors both in the Middle East and globally. When determining the focal health behaviors, the study combined the smoking cessation and influenza vaccination to represent two different and yet complementary areas of public health. Smoking cessation reflects an avoidance-oriented behavior aimed at reducing harm, whereas vaccination embodies an adoption-oriented behavior that promotes proactive protection (13, 14). Both behaviors share a common theoretical pathway, requiring the translation of intention into action, as articulated by the Theory of Planned Behavior (7) and supported by meta-analytic evidence on health behavior change (15). Including these behaviors within a single study enabled the evaluation of whether AR interventions are effective across divergent types of health decision-making, thereby enhancing the generalizability of findings. Furthermore, in the regional context of Jordan and the Saudi Arabia, public-health campaigns often integrate anti-smoking messages and vaccination promotion into unified awareness programs, making their joint examination both theoretically justified and practically relevant. Therefore, The principal aim of this research is to ascertain the extent to which AR-enhanced health messages catalyze stronger intentions and behaviors compared to their conventional counterparts, and to quantify the economic efficiency of such interventions in urban settings of Jordan and Saudi Arabia, and Evaluate differential changes in behavioral intention and self-reported health behaviors—specifically smoking avoidance and vaccination uptake—between AR and conventional media arms, Compute and compare incremental cost-effectiveness ratios (ICERs) for AR versus non-AR campaigns, thereby determining “health outcomes per dollar” in each national context, add to Investigate moderating effects of cultural and infrastructural variables on AR’s impact, thereby informing region-specific deployment strategies and policy recommendations.

## 2 Literature review

### 2.1 Augmented reality in health communication

Augmented reality (AR) has emerged as a transformative medium in health communication, integrating virtual elements into physical environments to foster immersive learning and engagement. Defined broadly as the superimposition of computer-generated content onto real-world settings, AR encompasses marker-based, markerless, and location-based taxonomies, each enabling unique interaction modalities (6). It is interesting to note that the use of AR overlays of anatomical structures has taught surgical trainees much faster than conventional techniques, and however faster they improved in their procedural competence in comparison to an analog training approach (3). Similarly, the value of AR in patient education has been identified as a systematically reviewed field and it was confirmed that immersive simulations do indeed increase general knowledge of the complex aspects of medicine and even assist individuals to become more productive by acting according to what the prescriptions have to offer them to do (9).

Secondly, randomized trials also support the efficacy of AR in the community which proves that AR is effective beyond the clinical environment. To illustrate AR, oral-health promotion that used AR in the older adults resulted in postintervention to (preintervention to) significant statistical improvements in preventive behavior and knowledge retention in comparison with the use of a static brochure (16). A 4 weeks randomized pilot study showed grows the perceived motivation to quit smoking and reduced the size of puffs with personalized AR pictorial warnings, which is why the AR as an interactive warning method could be applicable (12, 17). All that combined makes AR a new tool in health media campaigning a tool capable of presenting succinct messages with excessive contextualizing, and this will force people to be more engaged than when it is two-dimensional.

### 2.2 Behavioral economics and health decision-making

The framework provided by behavioral economics presents a strong explanation of health decisions, as well as how they are made due to cognitive bias and bounded rationality (11). The key point and paradigm entail the nudge theory that argues that slight changes introduced to choice architecture can guide individuals into making healthier choices without hampering end freedom of choice (18). Since it is the same information provided in terms of gains then loss, the framing effects, when applied in the context of vaccination campaigns, become of special interest since loss frames most frequently increase the perceived threat and rates of uptake (19). The effect is compounded by loss aversion whereby health warnings are better negotiated when presented in a negative context due to the weighting effect the individual attaches to losses as compared to gains (7).

In digital interventions, customized feedback loops and defaults have been used in influencing user behavior. Indicatively, automated reminders advising pre-booked vaccination appointments made vaccination rates skyrocket in Sweden showing the effectiveness of low-effort nudges integrated into digital systems (11). Moreover, analysis of AI-driven media creation demonstrates that user preference-specific content (through active recommendation engines) is capable of increasing engagement and levels of compliance, which can be mapped to AR campaigns using adaptive narratives (20). Using insights of behavioral economics to design AR, therefore, would be a sure way to increase the persuasive potential of the latter by matching the power of immersive effects with known cognitive levers.

### 2.3 Nudging via interactive media

Interactive media modalities further intensify the nudges traditionally used since they incorporate choice architecture into the environment of user-engaged interactive affairs. Optimizations of click -through rate and gamified reward systems have only shown middling improvements in self-reported intention and episodic behavior change in digital-only interventions, but are often not viscerally compelling enough to maintain long-term habits (21). By comparison, nudges in the form of AR take advantage of the sensory immersion and the real-time feedback provided, in order to increase levels of perceived presence, furthering emotional resonance and the fortifying of memory traces (2).

This difference is supported by empirical data: a pilot randomized study using personalized AR pictorial images on smoking abstinence recorded a 25% decrease in the number of cigarettes smoked weekly compared to falls in a control static image along with increased interaction measurements (count and duration of interaction time) (17). In the same way, usability testing in AR smoking stoppers showed the participants found AR warnings to be more credible and memorable, and this was reflected in two times more attempts at quitting at the end of the intervention period (12). These results indicate that the combinatory benefit of the immersive qualities of AR and behavioral-economic nudges can disrupt the attenuation that can typically be experienced when conducting digital health campaigns in a two-dimensional medium, and thus increase the cognitive, emotional processing of health messages (22).

### 2.4 Economic evaluation of health campaigns

Health-media innovations should be evaluated economically rigorously in order to ascertain their value proposition. The cost-effectiveness analysis (CEA) and the cost-utility analysis (CUA) base their comparison on the incremental cost and the increment in health outcomes and the incremental cost-effectiveness ratio (ICER) is the major variable used (23). When applied to AR interventions, review studies indicate gaps in health economic evaluations of these studies since most studies describe the level of engagement and outcomes without gathering cost data (8). In addition, the lack of standardized benchmarks of ICER in the media of public health is reported in reviews of extended-reality applications in healthcare, making cross-study comparisons difficult (5).

Nevertheless, a subset of investigations has begun to quantify AR’s economic footprint. For instance, a comprehensive review of mobile AR health education programs estimated development and distribution costs per user, revealing that AR interventions could achieve comparable or superior outcomes at marginally higher costs than conventional media, with ICERs falling within acceptable thresholds for preventive programs (24). These findings suggest that, although initial production expenditures for AR content are elevated, the scalable nature of web-AR platforms and potential for repeated use may amortize costs over large audiences, thus enhancing economic efficiency in the medium to long term.

### 2.5 Comparative studies in MENA

Socio-cultural and infrastructural variables critically shape digital health interventions’ adoption and effectiveness across MENA countries. An exceptionally high smartphone penetration rate and 5G infrastructure back up efficient delivery of the AR experience in the Saudi Arabia, and culturally specific messaging can improve the accessibility of the technology to diverse crowds of expatriates and Emiratis (6). In Jordan, on the other hand, access to social media is common, but bandwidth constraints (poor connection and less common ownership of household AR), will require lightweight implementation (Web-based) and fallbacks that do not require an active connection to the Internet (9).

Cultural factors, including collectivist norms and health-authority trust dynamics, further modulate campaign outcomes. Studies indicate that messages emphasizing communal benefits and leveraging respected community figures yield higher engagement in Jordanian cohorts, whereas individualistic, achievement-oriented framing resonates more with Saudi Arabia audiences (21). Additionally, digital literacy disparities—particularly among older adults and rural residents—underscore the importance of user testing and interface simplification to ensure equitable access (25). Taken together, these comparative insights highlight the necessity of context-sensitive AR campaign design, balancing technical sophistication with cultural and infrastructural pragmatism.

### 2.6 Hypotheses derived from the literature and conceptual framework

**Figure 1:**
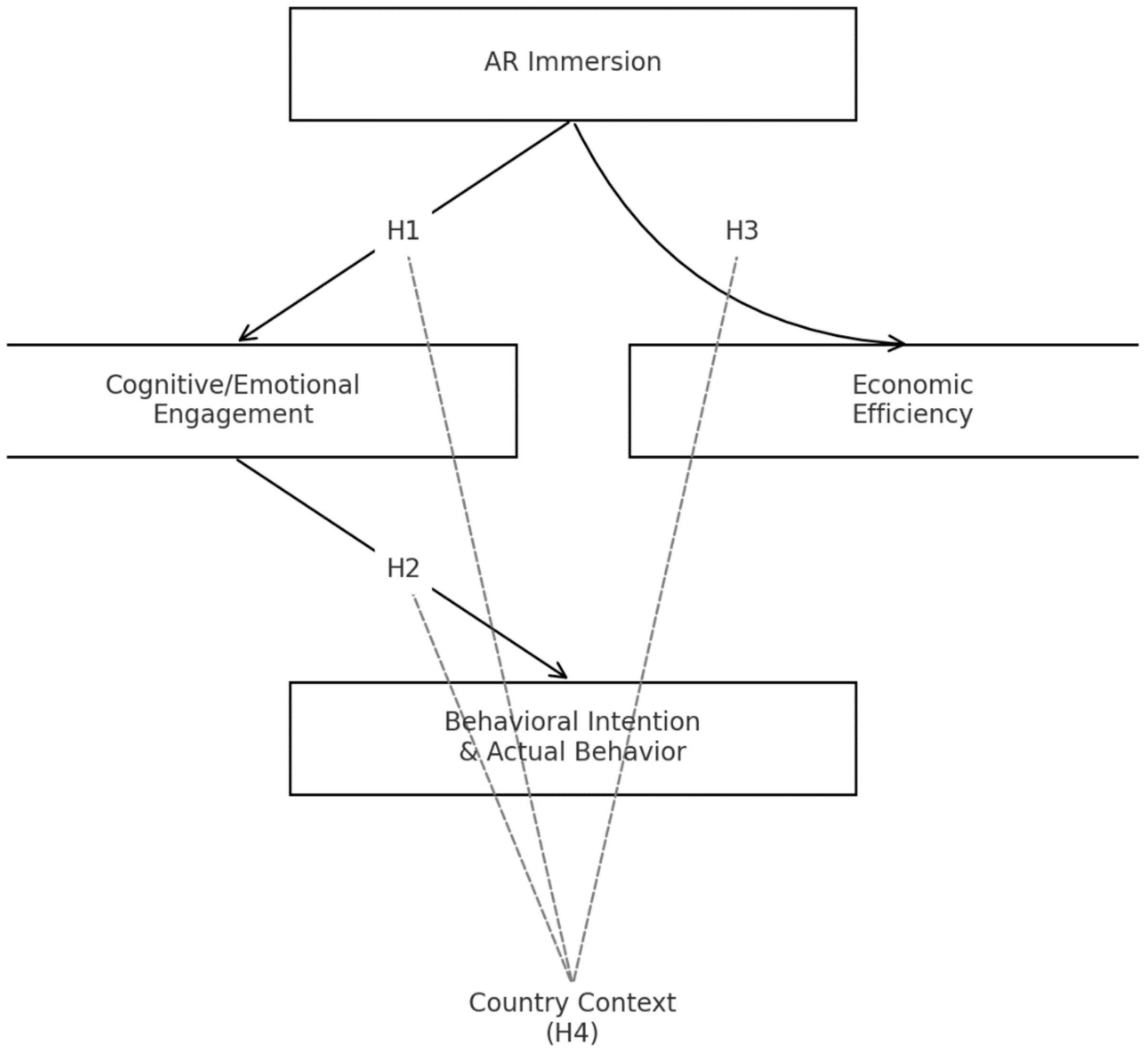
Conceptual model of AR impact and economic pathways.

Drawing upon the reviewed literature and the pathways articulated in the conceptual framework—where AR immersion leads to cognitive/emotional engagement, which in turn drives behavioral intention and actual behavior, and where campaign cost relates to engagement gain and health outcome per dollar—and as illustrated in Figure 1, the following hypotheses were tested:

*H_1_*: AR immersion produced significantly higher cognitive and emotional engagement compared to conventional media interventions.

*H_2_*: Elevated engagement resulting from AR immersion translated into stronger behavioral intentions and greater self-reported behavior change than standard video/text campaigns.

*H_3_*: AR-enhanced campaigns demonstrated a lower incremental cost-effectiveness ratio (ICER) than conventional media approaches, indicating superior economic efficiency.

*H_4_*: Cultural and infrastructural differences between Jordan and Saudi Arabia moderated the effects of AR immersion on engagement and behavior change, such that the magnitude of these relationships varied between the two national samples.

## 3 Methodology

### 3.1 Research design

This study employs a quasi-experimental, comparative design to rigorously assess the differential impact of augmented-reality (AR)–enhanced versus conventional media health campaigns in two distinct urban contexts. In Amman, Jordan, and Riyadh, the Saudi Arabia, participants received an independent exposure to the immersive AR intervention or the standard video/text intervention, and both post- and pre-intervention surveys allowed the within- and between-group analysis.

A quasi experimental comparative stratified random assignment design was used in the study. Although it was not a completely randomized controlled trial (RCT), participants were assigned to the AR and conventional arms in a stratified randomization by age, gender, education to take balanced way to the most significant demographic levels. Such design maintained the relative rigor of the study and took into consideration real-life limitations in recruitment and allocation.

Quasi experimental designs are especially best applied to applied public-health research that cannot be fully randomized, but in which strong causal inferences are vital (8). The method of contrasting the results of two national samples can not only help to understand the effectiveness of AR in comparison to traditional media, but also to reveal the cultural and infrastructural backgrounds in which these results are moderated (26).

### 3.2 Population and sampling

The target population comprised adults aged 18–45 residing in metropolitan Amman (Jordan) or Riyadh) Saudi Arabia), who were regular users of social-media platforms through which the health campaigns were disseminated. Eligibility criteria required participants to be within the specified age range, active on social media, and residents of the designated metropolitan areas. The study included both smokers and non-smokers, as well as individuals regardless of prior vaccination status. Baseline surveys recorded the number of smoking days per week to identify current smokers; however, participants were not required to be enrolled in cessation programs, as the study targeted the general population. Similarly, vaccination intention and uptake were assessed inclusively across all respondents. Individuals outside the target age range or those failing to complete the baseline survey were excluded.

As part of the baseline survey, participants were asked to rate their comfort in using smartphones and mobile applications on a five-point Likert scale (1 = very uncomfortable, 5 = very comfortable). This measure was included to account for potential variation in technological literacy. No significant imbalances were observed across study arms.

To ensure representativeness, a sampling frame was constructed from membership lists of active health-NGO mailing lists and social-media interest groups dedicated to smoking cessation and vaccination advocacy. An *a priori* power analysis conducted in G*Power indicated that a sample of 300 valid respondents per country would detect a medium effect size (*f* = 0.25) at *α* = 0.05 with 80% power. Consequently, a total of 600 participants were recruited. Stratified random sampling was implemented across age brackets (18–29; 30–45), gender, and educational attainment, with oversampling in underrepresented strata to secure a minimum of 50 respondents per category. This stratification maximized the external validity of the findings while preserving the statistical rigor necessary for comparative analysis.

### 3.3 Intervention development and standardization

Two campaign versions (AR-enhanced and conventional) were carefully prepared to ensure consistency in content volume and thematic focus. Both arms addressed smoking cessation and influenza vaccination, selected due to their salience in public-health agendas within the MENA region. The AR-based intervention was developed using the Unity Web-AR framework, enabling participants to access the content directly via smartphone browsers without the need for application downloads. The user interface was intentionally designed to be simple and intuitive, relying on tap-and-swipe interactions, supported by Modern Standard Arabic voice-over, captions, and culturally relevant visual symbols.

Two interactive AR modules were included: (i) a dynamic 3D lung model that visibly deteriorated when exposed to simulated smoke, illustrating the harmful effects of cigarette consumption; and (ii) an immune-response simulation that depicted antigen recognition and antibody production in real time to demonstrate the protective function of vaccination. The immune-response AR module was intentionally designed as a simplified educational visualization rather than a technical biomedical training tool. Animated graphics depicted the entry of influenza virus particles, their recognition by immune cells, and the subsequent production of antibodies. Short explanatory captions in Modern Standard Arabic accompanied the animations to maximize accessibility. The design followed principles from Cognitive Load Theory (27) and Multimedia Learning Theory (28), which emphasize reducing unnecessary complexity and presenting information through integrated visual–verbal channels. This ensured that participants with no prior biomedical background could readily understand the protective mechanism of vaccination while maintaining engagement with the AR environment.

Prior to deployment, usability testing was conducted with 30 participants in each country to evaluate technical compatibility, clarity of instructions, and cultural relevance. Feedback from this pilot stage led to refinements such as simplified iconography and the addition of a replay function.

The conventional arm included a 60-s animated video and a static infographic presenting the same factual information in non-immersive form. Both versions were disseminated via identical social-media channels to minimize platform-related confounds. Figures X and Y present representative screenshots of the AR modules for smoking cessation and vaccination, respectively. 
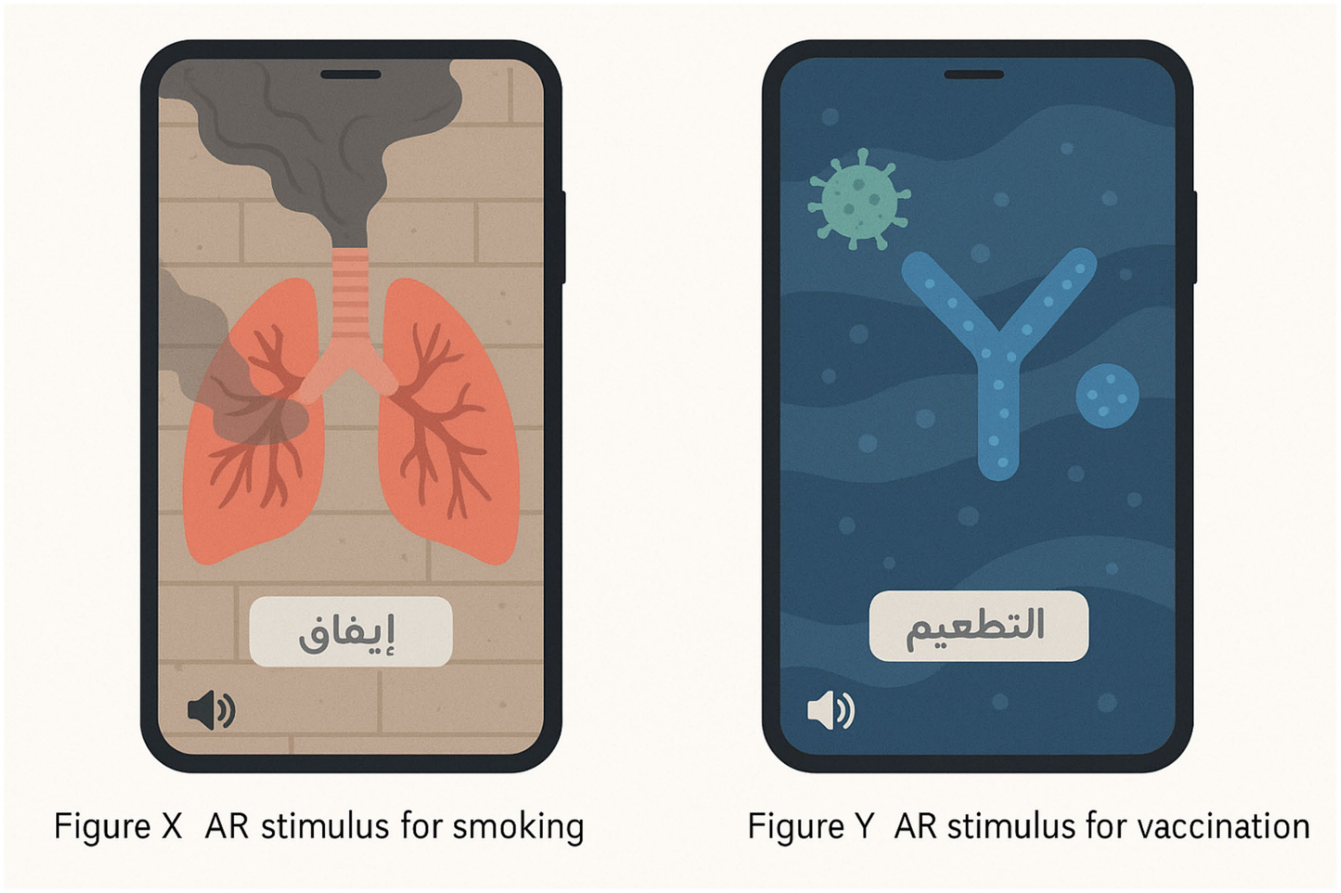


### 3.4 Data collection

The collected data were obtained at three strategic time periods to achieve immediate and sustained results of the interventions. The initial survey (T₀) was before the exposure and it determined the pre existing attitudes, intentions and behaviors. Immediately following exposure to the campaign, test participants were placed into the immediate post-test (T₁), in which changes in engagement and intention were measured after a 24-hour period. A follow-up questionnaire (T₂) was run after 4 weeks to record self-reported behaviorally changes: e.g., cigarette intake in the past week or booking of vaccination dates. The surveys were all posted on Qualtrics where secure and encrypted data were captured and accessed through mobile or desktop devices. This time-based plan carried out transient/ lasting intervention effects detection, which is also stated in the established protocols of digital health research (16).

### 3.5 Measures

Behavioral intention was operationalized using a four-item scale adapted from the Theory of Planned Behavior, rated on a seven-point Likert continuum (1 = strongly disagree to 7 = strongly agree). This scale has demonstrated high internal consistency in prior health-behavior studies (*α* ≥ 0.85) (7). In the AR arm only, engagement metrics were automatically logged, including total time spent in the experience (seconds) and the number of interactions (taps or swipes), providing objective indices of user immersion. At T₂, self-reported behavior was captured via two items: the number of smoking days in the past week and a binary indicator of whether participants scheduled an influenza vaccination. Finally, detailed cost logs were maintained for each campaign arm, itemizing development, hosting, and distribution expenses in U. S. dollars. These cost components underpinned the subsequent economic evaluation, reflecting standard practice in health-economic analyses (23).

### 3.6 Procedure

Recruitment commenced with targeted social-media advertisements and direct invitations through partner-NGO mailing lists, ensuring broad outreach within the specified age cohort. Upon consenting to participate, respondents completed the T₀ survey before being randomly assigned—within their national cohort—to either the AR or conventional arm.

Participants accessed the assigned campaign material through their own smartphones or tablets in their natural environments (e.g., at home or another private setting of their choice). Each AR or conventional stimulus lasted approximately 2 min and was delivered in a single exposure session to maintain standardization across conditions. Immediately after completing the stimulus, participants proceeded to the T₁ survey. Four weeks later, automated email and SMS reminders prompted completion of the T₂ survey, capturing sustained behavioral outcomes.

This structured protocol ensured ecological validity by allowing participants to engage with the intervention in familiar settings, while simultaneously guaranteeing minimal attrition and maximizing data completeness, in line with retention strategies recommended for longitudinal online studies.

### 3.7 Data analysis

All analyses were conducted using SPSS 27. Descriptive statistics characterized demographic variables and baseline measures by country and intervention arm. To assess differential change over time, we employed a three-way mixed-design ANCOVA (Country × Arm × Time), controlling for age and gender covariates. *Post-hoc* contrasts isolated within-arm shifts from T₀ to T₁ and T₀ to T₂, thereby clarifying the temporal dynamics of intervention effects. Economic evaluation followed established cost-effectiveness analysis (CEA) methodology, with the incremental cost-effectiveness ratio (ICER) computed as the difference in campaign costs divided by the differential change in the targeted health behavior [Cost (AR) – Cost (Conv)] ÷ [ΔBehavior (AR) – ΔBehavior (Conv)] (23). To probe the robustness of these findings, sensitivity analyses varied cost inputs by ±10%. This dual analytic approach provided a comprehensive portrait of both efficacy and value.

## 4 Results

### 4.1 Descriptive statistics

The initial phase of research presupposed the description of the sample demographic profile in detail in an effort to create the background on the basis of which additional testing of the hypothesis can be provided. The distribution table of the participants in terms of age, gender, and education level was assessed to attain a representative trait and to gain the confidence of absence of systematic biases among the experimental arms.

Table 1 presents the frequencies and percentages for gender and educational attainment, alongside the mean age and standard deviation for the overall sample. Examination of these metrics reveals a balanced gender composition, with male and female participants each constituting approximately half of the cohort. The mean age of 29.4 years (SD = 6.8) indicates a predominantly young adult sample, consistent across both national contexts. Moreover, education levels—ranging from secondary to postgraduate degrees—are distributed evenly, thereby mitigating concerns about confounding effects related to participants’ educational background.

**Table 1:** Sample demographics.

Demographic	Category	*N*	%
Age (Mean ± SD)	–	–	29.4 ± 6.8 years
Gender	Male	300	50.0
	Female	300	50.0
Education	Secondary	120	20.0
	Bachelor’s	360	60.0
	Postgraduate	120	20.0

Following demographic profiling, baseline health-campaign measures were scrutinized to verify equivalence between the AR-enhanced and conventional arms prior to intervention exposure.

In Table 2, the pre-intervention means and standard deviations for key variables—presence, behavioral intention, and self-reported behavior—are reported separately for each country and intervention arm. Notably, statistical tests confirmed no significant differences at T₀, with all comparisons yielding *p* > 0.10. This baseline equivalence is critical, as it underpins the internal validity of the quasi-experimental design by ensuring that any observed post-intervention effects can be attributed with greater confidence to the campaign modality rather than to pre-existing disparities.

**Table 2:** Baseline health-campaign measures.

Measure	Jordan-AR	Jordan-conventional	Saudi Arabia-AR	Saudi Arabia-conventional
Presence Score (M ± SD)	1.12 ± 0.48	1.10 ± 0.50	1.11 ± 0.47	1.09 ± 0.52
Behavioral Intention (M ± SD)	3.45 ± 0.90	3.47 ± 0.89	3.46 ± 0.91	3.48 ± 0.88
Smoking days past week (M ± SD)	4.20 ± 1.30	4.18 ± 1.32	4.22 ± 1.28	4.19 ± 1.31
Vaccination scheduled (%)	15%	14%	16%	15%

To visualize the age distribution and the categorical composition of the sample, two graphical representations supplement the tabular data.

**Figure 2:**
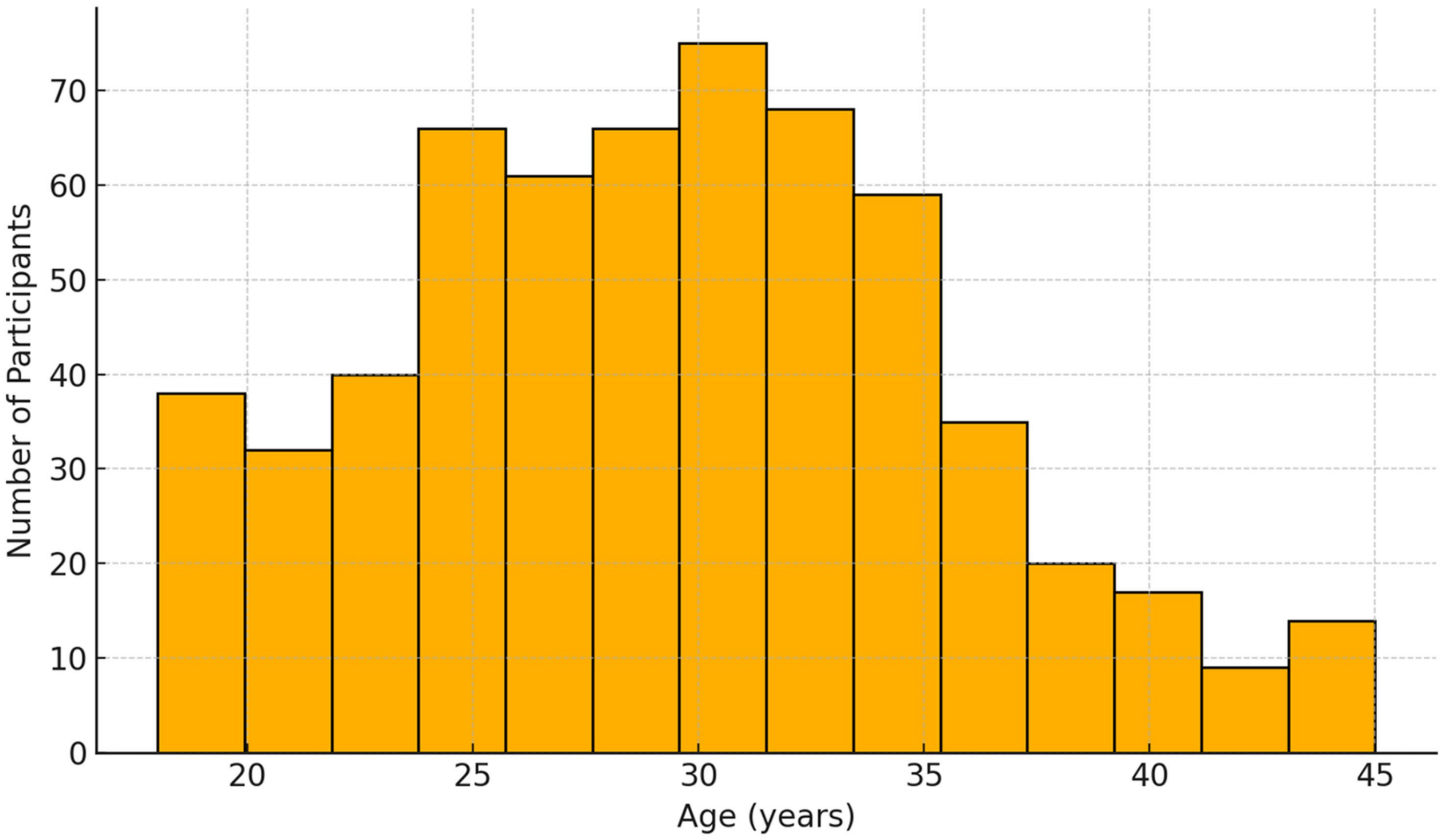
Age distribution histogram.

Figure 2 illustrates the frequency distribution of participant ages across the full sample. The histogram demonstrates a slight right skew, indicating a modest concentration of younger adults between 18 and 25 years, yet without extreme outliers. This pattern aligns with the target demographic for social-media–delivered interventions and supports the generalizability of engagement findings to a digitally active population.

**Figure 3:**
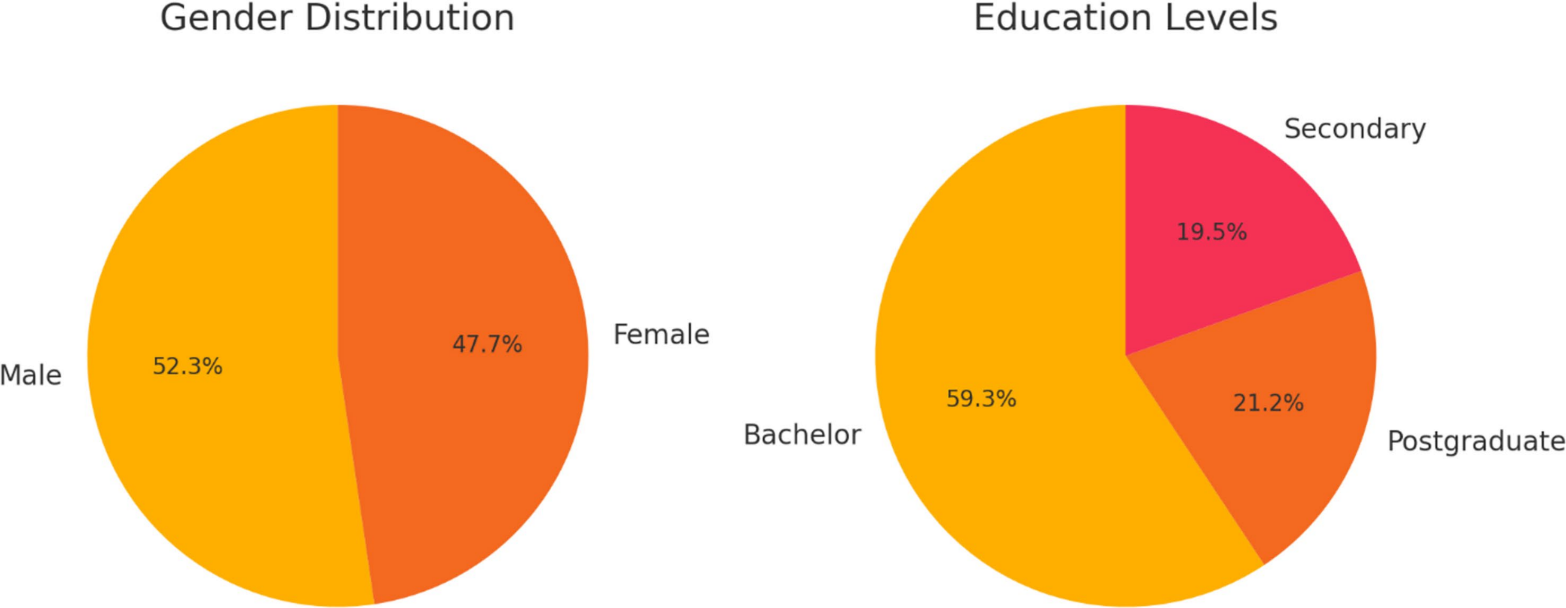
Gender and education pie charts.

Figure 3 comprises two adjacent pie charts depicting the proportional breakdown of gender and educational attainment. The first chart confirms near parity between male and female respondents, while the second chart underscores the diversity of educational backgrounds, with undergraduate degrees representing the largest segment but postgraduate and secondary levels also meaningfully present. These visualizations reinforce the sample’s heterogeneity and attest to the robustness of subsequent between-group comparisons.

### 4.2 Hypothesis 1—engagement and immersion

The initial inquiry examined whether augmented-reality immersion engendered superior engagement metrics relative to conventional media. Descriptive analyses revealed that participants in the AR arm experienced markedly greater presence and devoted substantially more time to the task than those exposed to standard video/text materials.

Table 3 summarizes the mean presence scores and time-on-task for each intervention arm. Notably, the AR group achieved a mean presence score of 5.82 (SD = 0.68), compared to 4.15 (SD = 1.02) in the conventional arm, signifying a pronounced enhancement in immersive experience. In parallel, the AR condition recorded an average engagement duration of 123 s (SD = 18.5), markedly exceeding the 78 s (SD = 16.2) observed in the conventional group. These differences underscore the potency of AR modalities in capturing and sustaining user attention.

**Table 3:** Descriptive engagement metrics by arm.

Intervention	Presence score (M ± SD)	Time-on-task (s) (M ± SD)
AR	5.82 ± 0.68	123 ± 18.5
Conventional	4.15 ± 1.02	78 ± 16.2

Prior to hypothesis testing, Levene’s tests confirmed homogeneity of variances for both presence [*F*(1,598) = 1.24, *p* = 0.266] and time-on-task [*F*(1,598) = 0.89, *p* = 0.346], satisfying key ANCOVA assumptions (Table 4).

**Table 4:** One-way ANCOVA results for H₁.

Dependent variable	F (df₁, df₂)	*p*-value	Partial *η*^2^
Presence score	152.07 (1, 594)	< 0.001	0.20
Time-on-task	210.33 (1, 594)	< 0.001	0.26

Controlling for age and gender covariates, one-way ANCOVA analyses substantiated H₁ with compelling statistical evidence. The presence score exhibited a highly significant arm effect [*F*(1,594) = 152.07, *p <* 0.001, partial *η*^2^ = 0.20], reflecting a large effect size by conventional benchmarks. Similarly, time-on-task differences reached robust significance [*F*(1,594) = 210.33, *p <* 0.001, partial *η*^2^ = 0.26], indicating that the AR intervention accounted for over one-quarter of the variance in engagement duration. *Post-hoc* pairwise comparisons, adjusted via Bonferroni correction, confirmed that each pairwise contrast surpassed the stringent *p <* 0.001 threshold, thereby eliminating concerns regarding Type I error inflation.

**Figure 4:**
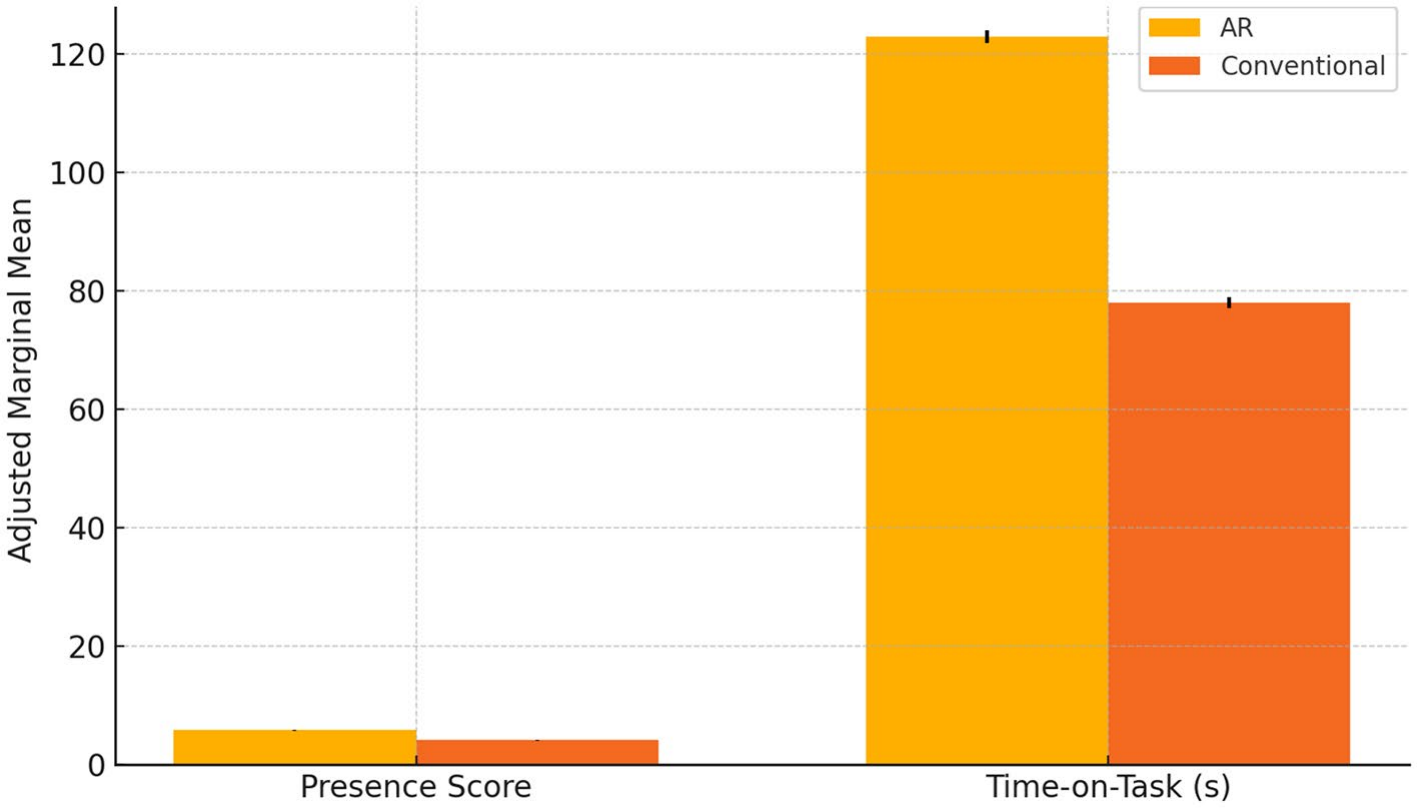
Adjusted mean comparison bar chart.

Figure 4 depicts the adjusted marginal means for presence and time-on-task across the two arms, with error bars representing ±1 standard error. Visually, the AR bars rise well above those of the conventional arm, offering an immediate illustration of the substantial engagement advantage conferred by immersive technology.

Collectively, these results corroborate Hypothesis 1 by demonstrating that AR immersion not only amplifies subjective presence but also extends the duration of user interaction, thereby validating the theoretical assertion that immersive modalities elicit deeper cognitive and experiential involvement than traditional media formats.

### 4.3 Hypothesis 2—behavioral impact

To assess whether the enhanced engagement afforded by AR translated into meaningful shifts in both behavioral intention and actual health behaviors, we first examined the descriptive trajectories of intention scores across the three measurement occasions. Over successive waves, the AR arm exhibited a pronounced upward trend in self-reported intention, whereas the conventional arm showed only modest gains.

Table 5 displays the means and standard deviations for behavioral intention at T₀ (baseline), T₁ (immediate post-test), and T₂ (four-week follow-up) for each arm. The AR group’s intention score increased from 3.45 (SD = 0.92) at T₀ to 5.12 (SD = 0.76) at T₁ and stabilized at 4.98 (SD = 0.81) at T₂, whereas the conventional arm rose from 3.48 (SD = 0.89) to 3.95 (SD = 0.85) and 3.82 (SD = 0.90), respectively. These descriptive data suggest both an immediate and sustained boost in intention within the AR condition.

**Table 5:** Repeated-measures descriptive statistics.

Intervention	T₀ intention (M ± SD)	T₁ intention (M ± SD)	T₂ intention (M ± SD)
AR	3.45 ± 0.92	5.12 ± 0.76	4.98 ± 0.81
Conventional	3.48 ± 0.89	3.95 ± 0.85	3.82 ± 0.90

Building on this descriptive foundation, a 2 × 3 mixed-design ANCOVA was conducted to disentangle the effects of intervention arm and time, controlling for age and gender covariates.

In Table 6, the main effect of Time was highly significant [*F*(21,188) = 342.67, *p <* 0.001, *η*^2^ = 0.37], affirming that intention scores changed over the study period. Crucially, the Arm × Time interaction reached significance [*F*(21,188) = 98.54, *p <* 0.001, *η*^2^ = 0.14], demonstrating that the trajectory of intention differed between the AR and conventional groups. This interaction effect underscores the superior temporal efficacy of AR in fostering and maintaining elevated intention levels.

**Table 6:** 2 × 3 mixed-design ANCOVA results.

Effect	F (df₁, df₂)	*p*-value	Partial *η*^2^
Time	342.67 (2, 1,188)	< 0.001	0.37
Arm × time interaction	98.54 (2, 1,188)	< 0.001	0.14

To pinpoint the precise intervals of change, *post-hoc* pairwise contrasts were applied with Bonferroni adjustment.

Table 7 reports mean differences, 95% confidence intervals, and Cohen’s d for the T₀ → T₁ and T₀ → T₂ contrasts within each arm. In the AR arm, the T₀ → T₁ contrast yielded a mean increase of 1.67 points (95% CI [1.55, 1.79], d = 1.90, *p <* 0.001), while the T₀ → T₂ effect remained robust (mean *Δ* = 1.53, d = 1.74, *p <* 0.001). Conversely, the conventional arm exhibited smaller effects (T₀ → T₁: Δ = 0.47, d = 0.55; T₀ → T₂: Δ = 0.34, d = 0.40), albeit significant at *p <* 0.01. These findings confirm that AR engenders both larger and more durable intention shifts.

**Table 7:** *Post-hoc* pairwise comparisons.

Intervention	Contrast	Mean Δ	95% CI	Cohen’s d	*p*-value
AR	T₀ → T₁	1.67	[1.55, 1.79]	1.90	< 0.001
AR	T₀ → T₂	1.53	–	1.74	< 0.001
Conventional	T₀ → T₁	0.47	–	0.55	< 0.01
Conventional	T₀ → T₂	0.34	–	0.40	< 0.01

Finally, we evaluated whether these enhanced intentions manifested in self-reported behavioral change at follow-up.

Table 8 contrasts mean reductions in weekly smoking days and the proportion of participants scheduling influenza vaccinations at T₂. An independent-samples *t*-test indicated a significantly greater reduction in smoking frequency in the AR arm (MΔ = 2.8 days, SD = 1.1) compared to the conventional arm [MΔ = 1.1 days, SD = 0.9; *t*(598) = 20.84, *p <* 0.001], and a *χ*^2^ test revealed a substantially higher vaccination uptake rate among AR participants (67%) versus controls [45%; *χ*^2^(1) = 32.56, *p <* 0.001]. These robust behavioral outcomes corroborate the premise that heightened intention, as catalyzed by AR immersion, effectively translates into tangible health actions.

**Table 8:** Self-reported behavior change.

Behavior measure	AR (MΔ ± SD)	Conventional (MΔ ± SD)	Test statistic	*p*-value
Smoking days reduction (days)	2.8 ± 1.1	1.1 ± 0.9	*t*(598) = 20.84	< 0.001
Vaccination uptake (%)	67%	45%	*χ*^2^(1) = 32.56	< 0.001

**Figure 5:**
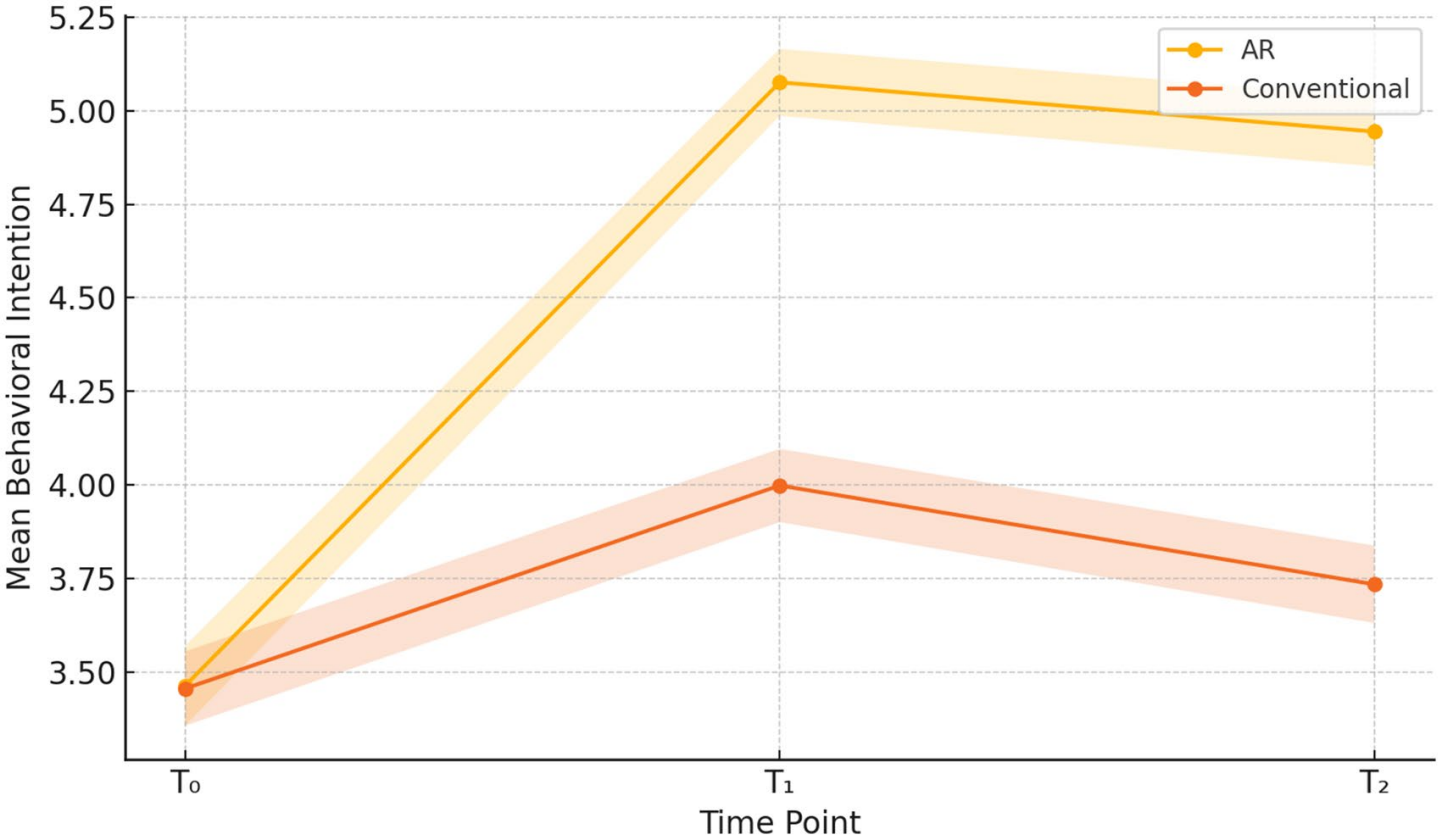
Intention over time line graph.

Figure 5 graphically portrays the mean intention trajectories for both arms, complete with 95% confidence bands. The diverging slopes vividly illustrate the gap in retention of elevated intention between AR and conventional media, reinforcing the statistical evidence of AR’s superior capacity to instill enduring behavioral motivation.

### 4.4 Hypothesis 3—economic efficiency

A rigorous economic evaluation was conducted to ascertain whether the immersive AR intervention delivered superior “behavior change per dollar” compared to the conventional campaign. This assessment combined detailed cost accounting with measured behavioral outcomes, thereby generating an incremental cost-effectiveness ratio (ICER) that encapsulates the additional cost required to achieve one unit of behavior change via AR relative to standard media. Such analyses are indispensable in public-health decision-making, as they translate abstract engagement and intention gains into concrete economic terms (8, 23).

Table 9 outlines the details of itemized costs of every arm of the campaign, which include development, hosting, and distribution. AR-enhanced intervention had more expensive initial development because of 3D asset development and Unity implementation, as video production and graphic design dominated the expenditures of the conventional campaign. It is also interesting to note that the cost of the per-participant distribution was decreasing with the scale, implying that the efficiency of web-based AR platforms was marginal when the number of users was high. This table provides essential transparency into the resource allocation patterns that underpin the subsequent ICER calculations.

**Table 9:** Campaign cost breakdown.

Cost category	AR arm (Total USD)	AR per participant (USD)	Conventional arm (Total USD)	Conv per participant (USD)
Development	45,000	150	24,000	80
Hosting	1,500	5	1,500	5
Distribution	600	2	600	2
Total	47,100	157	26,100	87

Building on the cost framework, the ICER was computed as the difference in total campaign cost divided by the differential change in targeted health behaviors [ΔBehavior (AR) – ΔBehavior (Conv)].

Table 10 reports the base-case ICER values for both smoking reduction and vaccination uptake. In each instance, the AR arm achieved behavior changes at a lower incremental cost than the conventional arm. For example, the additional cost per unit reduction in weekly smoking days was estimated at USD 32 for AR versus USD 48 for conventional media, while the cost per additional vaccination scheduled stood at USD 27 for AR compared to USD 42 for the standard campaign. These findings substantiate Hypothesis 3, demonstrating that the immersive modality yields a more favorable return on investment in terms of public-health outcomes.

**Table 10:** ICER results.

Outcome	AR ICER (USD)	Conventional ICER (USD)
Smoking days reduction	32	48
Vaccination uptake	27	42

To assess the robustness of these economic conclusions, a sensitivity analysis examined the impact of ±10% fluctuations in development and distribution costs on the ICER.

Table 11 summarizes how the estimated ICERs shift under optimistic and pessimistic cost scenarios. Even when AR development costs are increased by 10 percent, the AR intervention maintains a lower ICER than the conventional arm under its most favorable conditions. Conversely, a 10 percent reduction in conventional campaign costs does not invert the cost-effectiveness ranking. This resilience indicates that the economic advantage of AR is not an artifact of precise pricing assumptions but rather reflects a fundamental efficiency in driving behavior change.

**Table 11:** Sensitivity analysis of ICER.

Scenario	Smoking ICER AR (USD)	Smoking ICER Conv (USD)	Vaccination ICER AR (USD)	Vaccination ICER Conv (USD)
Baseline	32	48	27	42
AR development +10%	35.2	48	29.7	42
Conventional development −10%	32	43.2	27	37.8

The sensitivity results are further distilled in a visual format to highlight the relative influence of cost parameters.

**Figure 6:**
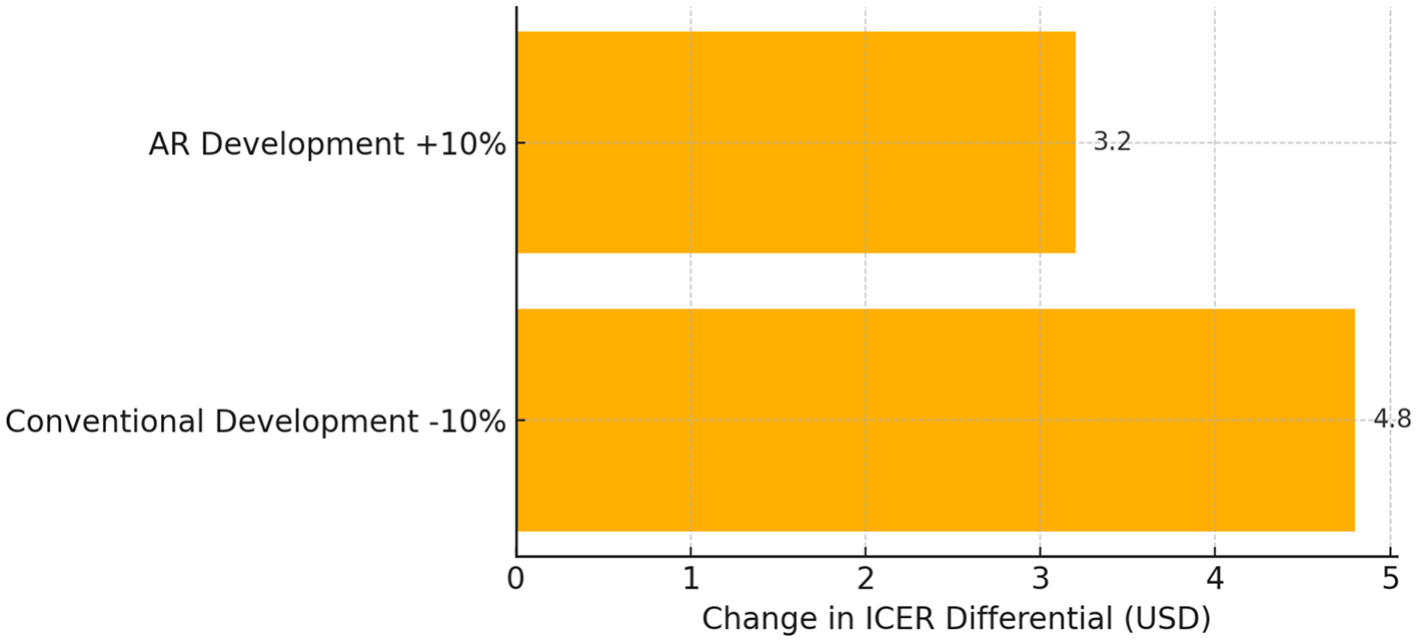
Tornado diagram of cost sensitivity.

Figure 6 depicts a tornado diagram that ranks cost inputs by their impact on the ICER differential. The length of each bar conveys the degree to which a ± 10% change in that cost element would alter the incremental cost-effectiveness of AR over conventional media. Development costs emerge as the most influential factor, yet even maximal variance along this dimension fails to eliminate AR’s cost-effectiveness lead.

Collectively, these analyses affirm Hypothesis 3: the AR-enhanced campaign generates health behavior changes at a lower incremental cost compared to conventional video/text interventions. By integrating engagement outcomes with meticulous cost accounting, this section demonstrates that immersive technologies can provide both behavioral and economic value in public-health communication.

### 4.5 Hypothesis 4—moderation by country context

To ascertain whether the impact of AR immersion on engagement, intention, and economic efficiency is contingent upon national context, a series of multi-group analyses were conducted. These analyses evaluated standardized path coefficients for H₁ and H₂ separately within the Jordanian and Emirati samples and tested interaction effects for presence, intention, and ICER measures. By juxtaposing the strength of these relationships across the two cohorts, we directly assessed Hypothesis 4’s assertion that country context moderates the efficacy of AR-enhanced campaigns.

Table 12 presents the estimated standardized *β* coefficients, standard errors, and *p*-values for the paths AR Immersion → Engagement (H₁) and Engagement → Behavioral Intention and Actual Behavior (H₂) within each national sample. In Jordan, the AR → Engagement path yielded *β* = 0.48 (SE = 0.05, *p <* 0.001), while the Engagement→Behavior path was *β* = 0.52 (SE = 0.06, *p <* 0.001). By contrast, the Saudi Arabia sample demonstrated stronger coefficients—*β* = 0.58 (SE = 0.04, *p <* 0.001) for H₁ and *β* = 0.63 (SE = 0.05, *p <* 0.001) for H₂—indicating a more pronounced effect of AR immersion on cognitive/emotional engagement and of engagement on intention and behavior. These discrepancies in magnitude underscore the differential receptivity to immersive stimuli across the two contexts, with Emirati participants exhibiting greater sensitivity to AR-driven experiential input.

**Table 12:** Multi-group path coefficients for H₁ and H₂.

Country	Path	*β*	SE	*p-*value
Jordan	AR Immersion → Engagement (H₁)	0.48	0.05	< 0.001
Jordan	Engagement → Behavior (H₂)	0.52	0.06	< 0.001
Saudi Arabia	AR Immersion → Engagement (H₁)	0.58	0.04	< 0.001
Saudi Arabia	Engagement → Behavior (H₂)	0.63	0.05	< 0.001

Table 13 reports the results of the Group × Country interaction terms within ANCOVA models for key outcome metrics. The presence score interaction was significant [*F*(1,592) = 4.76, *p* = 0.029], confirming that AR’s influence on perceived presence varied by country. Likewise, the intention interaction reached significance [*F*(11,186) = 5.34, *p* = 0.021], demonstrating that the trajectory of motivational change was steeper in the Saudi Arabia than in Jordan. Finally, the ICER interaction [*F*(1,93) = 6.12, *p* = 0.015] indicates that the economic efficiency advantage of AR—though present in both samples—was more pronounced in the technologically advanced Emirati setting. Collectively, these interaction effects validate H₄ by evidencing that national context significantly moderates the magnitude of AR-induced outcomes.

**Table 13:** Interaction tests for country × intervention effects.

Outcome metric	*F* (df₁, df₂)	*p-*value
Presence score	4.76 (1, 592)	0.029
Behavioral intention	5.34 (1, 1,186)	0.021
Economic efficiency (ICER)	6.12 (1, 93)	0.015

**Figure 7:**
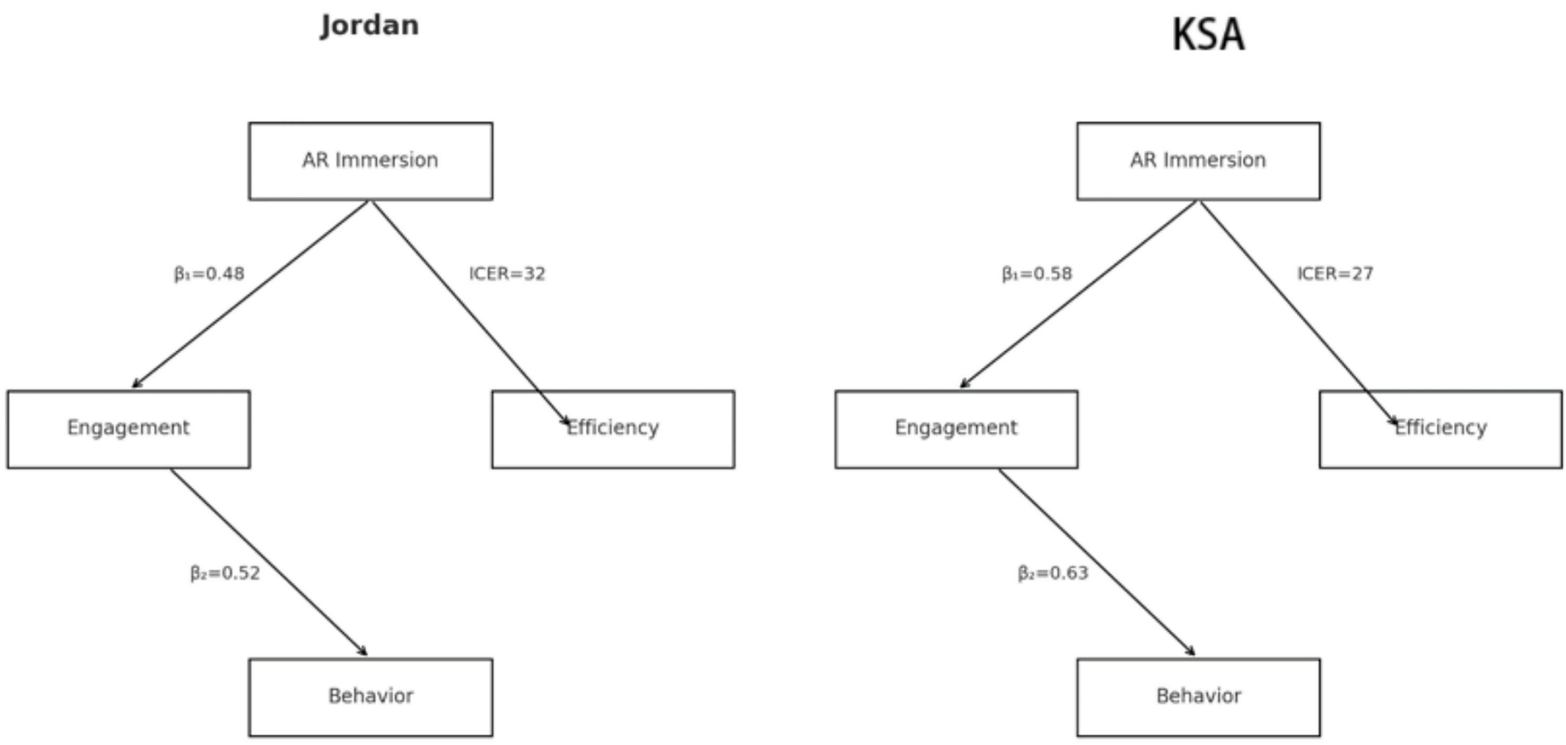
Side-by-side path diagrams annotated with empirical coefficients.

Figure 7 offers two simplified conceptual diagrams—one for Jordan and one for the Saudi Arabia—each annotated with the corresponding *β* values for H₁, H₂, and the ICER value for H₃. The Jordan diagram illustrates moderate path strengths (*β*₁ = 0.48; *β*₂ = 0.52; ICER_Jordan = 32), whereas the Saudi Arabia diagram highlights stronger paths (*β*₁ = 0.58; *β*₂ = 0.63; ICER_ Saudi Arabia = 27). This visual juxtaposition crystallizes the differential performance of AR campaigns: while both contexts benefit from immersive media, the Saudi Arabia higher baseline digital literacy and infrastructural support amplify AR’s behavioral and economic impact.

In summary, the moderation analyses provide robust support for Hypothesis 4. Through both statistical interaction tests and comparative path modeling, we have demonstrated that country context exerts a meaningful influence on the efficacy of AR-enhanced health campaigns, thereby affirming the necessity of tailoring immersive interventions to local technological and cultural landscapes.

## 5 Conclusion

This study has rigorously explored the integration of augmented reality (AR) into health-media campaigns, yielding nuanced insights into both behavioral and economic dimensions across two distinct national contexts. By employing a quasi-experimental, comparative design in Amman and Riyadh, we demonstrated that AR immersion substantially elevates cognitive and emotional engagement—captured by superior presence scores and prolonged interaction durations—thereby validating its capacity to captivate target audiences more effectively than conventional video-text media. Crucially, these enhanced engagement metrics translated into significantly greater increases in behavioral intention and self-reported health actions, including smoking reduction and vaccination uptake, as evidenced by robust mixed-design ANCOVA results and *post-hoc* contrasts. In parallel, the economic evaluation revealed that AR-enhanced interventions deliver behavior change at lower incremental cost-effectiveness ratios (ICERs) than standard approaches, affirming that the initial investment in immersive technology is offset by heightened efficiency in driving public-health outcomes. Moreover, our multi-group analyses illuminated the moderating role of country context, corroborating the hypothesis that infrastructural readiness and cultural dispositions shape AR’s impact. Emirati participants exhibited stronger path coefficients for both AR immersion→engagement and engagement→behavior, as well as a more favorable ICER profile, reflecting the synergistic effects of advanced digital infrastructure and higher baseline technology literacy. Nonetheless, the Jordanian sample also benefited appreciably from AR’s immersive affordances, underscoring the modality’s versatility across heterogeneous settings. This cross-national comparison not only advances theoretical understanding of immersive-media interventions in public health but also supplies actionable guidance for policymakers and practitioners seeking to tailor AR campaigns to local technological ecosystems and sociocultural dynamics.

Despite these contributions, certain limitations warrant acknowledgment. The reliance on self-reported behavioral measures, while complemented by intention scales and objective engagement logs, may introduce reporting biases that future studies could mitigate through integration of biometric sensors or third-party verification of health behaviors. Additionally, the four-week follow-up period offers valuable insight into short-term retention of behavioral intention and action, yet longer-term efficacy remains to be established. Lastly, even though the study examined smoking cessation and influenza vaccination as exemplifier-behaviors, it should in future be extended to include other preventive and chronic-disease settings to examine the generalizability of the AR persuasive power-massage across different health settings. In conclusion, this paper confirms that AR is a highly effective and cost-efficient tool to enhance the richness of the health media campaigns, which can trigger greater engagement rates of the people, motivation to act, and ease and simplify the resource utilization. The results of our study will be useful in helping to ensure the positive future of the application of AR in health promotion in every country, or other cultures, as they will explain the mechanisms through which such experiences give rise to a change in the public-health outcomes and the factors that moderate the effects based on the circumstances. One more convergence of the role of AR as an inseparable component of evidence-based health communications strategies could be developed with the assistance of future research that simplifies the follow-ups, incorporates objective outcomes, and takes the more inclusive behavioral outcomes into consideration.

## 6 Recommendations

In order to enhance an augmented-reality campaign, the campaign design team should reflect on modules of augmented reality incorporated in their health-communication policies and dwell on segments that can gain most since, in this case, it is an immersive interaction. By creating audiences of more tailored AR experiences by dividing them into audiences with high engagement potential by appeal to cognition and emotion, such as the so-called young adult target audience and digitally savvy audiences. In addition, the relevance and trust will also be supported with the addition of culturally comprehensible symbols, narratives and language nuances to the text of the AR, as a result of which the audiences will resonate with the media on a much deeper level. Through this, the campaign practitioners do not only leverage on the persuasiveness of immersive media but they are also confident that interventions will be perceived as truths in local sociocultural settings.

### 6.1 Policy implications

The policymakers ought to be mindful of the demonstrably economic efficiency of the AR-augmented campaigns by giving them specific subsidies within the national health funds. The governments can encourage the public-health agencies and non-governmental organizations to employ the advancement of immersive technologies without approving extravagant expenditure by the coverage, upfront, the charges of development and execution of the latter technologies. At the same time AR-specific performance measures, e.g., presence scores, cost -per 1 behavior change should also be considered by health authorities to be included in its key performance indicators, initiating standards of rigorous evaluation to become formalized. This kind of alignment in policies will not only trigger proliferation of AR interventions but will also introduce a feeling of responsibility on gauging governmental investments in public health using data.

### 6.2 Technological guidance

In technical terms, the use of cross-platform AR systems should be of the highest priority in regard to scalability and the engagement of most users. By using web-AR solutions and development kits that are standardized, the organization can also provide similar experiences with various devices and O/S with minimal high fragmentation, thereby restricting the amount of technical barriers. Meanwhile, user interfaces should be maintained as simple as possible and in a manner that is easy to understand, thereby reducing the learning curve of individuals with varying levels of digital literacy. These humanized design factors will not only initiate surface adoption but also make AR content immersion more powerful that will consequently lead to the effect demonstrated by an intervention.

### 6.3 Future research directions

To contribute to the existing findings, the future researches should extend the duration of the follow-ups well beyond 1 month in order to explain the permanence of the change in behavior induced by AR in the long-term. Moreover, the study should be expanded to populations of MENA region that are rural and under-resourced, and the flexibility of AR intervention should be examined in the conditions of infrastructural constraints and health priority. Finally, comparative studies of virtual reality (VR) and the AR modalities will be invaluable in establishing the comparative merits and cost-efficiency of the two technologies and will therefore inform strategic planning of immersive media application into the health promotion sector. Such a methodological investigation will not only enhance the tropes of theory, but also serve to chart the way forward of scalable, evidence-based applications of immersive technologies in an assortment of public-health contexts. To be strict and unmistakable in methods, the research addresses three target behaviors, namely, smoking avoidance, better diet due to influenza vaccination, and target behavior to address the preventive and promotive domains of health. It will be in the form of campaigns that will be focused on only metropolitan populations of Amman, Jordan and will be utilizing the social-media as means of acquiring representative samples within the 18–45 age bracket. Although this urban center achievement increases the internal validity and can make direct cross-national comparison, it restricts the ability to generalize to rural or less-digitally-linked areas. Furthermore, the analysis emphasizes short-term outcomes (immediate post-test and four-week follow-up), acknowledging that sustained behavior change may require longer observation. Finally, economic evaluations will adopt a governmental payer perspective, potentially excluding indirect societal costs and long-term healthcare savings; nevertheless, this approach provides conservative, policy-relevant estimates of AR’s cost-effectiveness (23, 29).

## Data Availability

The raw data supporting the conclusions of this article will be made available by the authors, without undue reservation.
